# A Computational Fluid Dynamic Analysis of Peri-Bracket Salivary Flow Influencing the Microbial and Periodontal Parameters

**DOI:** 10.1371/journal.pone.0062242

**Published:** 2013-04-19

**Authors:** Ping Zhu, Han Lin, Yi Han, Yi Lin, Yue Xu, Zhaoqiang Zhang

**Affiliations:** 1 Department of Orthodontics, Guanghua School of Stomatology, Hospital of Stomatology, Sun Yat-sen University, Guangdong Provincial Key Laboratory of Stomatology, Guangzhou, Guangdong, People’s Republic of China; 2 Department of Applied Mechanics and Engineering, Sun Yat-Sen University, Guangzhou, Guangdong, People’s Republic of China; 3 Department of Oral and Maxillofacial Surgery, Hospital of Stomatology, Guangzhou Medical College, Guangzhou, Guangdong, People’s Republic of China; UC Davis School of Medicine, United States of America

## Abstract

Fixed vestibular appliances decrease the “self-cleansing” action of saliva and promote aggregation of dental plaque by disturbing the salivary flow field on tooth surfaces, leading to a higher prevalence of enamel demineralization and periodontal diseases. In the current study, we investigated the salivary dynamic characteristics of plaque retention and periodontal status around appliances during orthodontic treatment. By reconstructing lower central incisors and orthodontic appliances, we simulated saliva flow on the tooth surface and then characterized and quantified the salivary flow pattern surrounding the bracket and archwire. In parallel, we tested the total peri-bracket bacterial counts and periodontal status to assess interrelations. Our results demonstrate that orthodontic appliances disturb the salivary flow field on tooth surfaces and can lead to a decrease in salivary velocity and an increase in bacterial numbers. Local vortexes forming in the areas gingival to the bracket, together with the narrow space limitation, contributed to the periodontal inflammatory response. This study confirms that changes in salivary flow are an obvious predisposing factor for bacterial accumulation, and advances the ability to replicate, *in* *vitro*, the salivary characteristics of plaque retention and periodontal status around appliances during orthodontic treatment.

## Introduction

Fixed vestibular appliances often increase the risk of both, enamel demineralization [1] and periodontal diseases [2], by promoting aggregation of dental plaque [3] locally as well as ectopically to less common sites on the labial surface of the teeth. A protective and nourishing community of periodontopathogenic bacteria [4], dental plaque is formed due to the dynamic interactions among diet, salivary flow, microorganisms and biochemical factors [5]. Bacterial adhesion to solid surfaces appears to be influenced by many factors such as “self-cleansing” mechanisms [6], movement of the oral musculature as well as saliva. Xerostomia and hyposalivation are prevalent in patients with Type 2 diabetes who have higher incidence of periodontitis [7], which is an issue of growing concern. The insertion of brackets impedes the removal of food debris [8] and simultaneously disturbs the normal distribution of salivary flow, thereby causing environmental changes in the microbiological flora. The increasing number of oral bacteria may account for rapid plaque growth and decrease in pH value [9], leading to continued structural damage to the teeth and their supporting tissue [10].

As a major clinical concern, demineralization and periodontal diseases in orthodontic subjects are considered to be inevitable and adverse problems correlated with the morphology of appliances, materials used and bonding locations, among others. Gastel [11] suggested that bracket design could have a significant impact on bacterial load and on periodontal parameters. Moreover, several studies regarding dental plaque formation and bracket placement, either incubated in artificial saliva [12] or immersed in unstimulated saliva [13] have been performed, where the capacity for biofilm formation varies with the various surface characteristics such as bracket roughness [14], ligation modes [15], and adhesives [16]. However, such systems are not representative of flowing saliva, but that of resting saliva containing chemical agents. The involvement of changes in salivary flow pattern in plaque retaining has not been realized.

A study utilizing techniques similar to *in* *vivo* saliva would be useful to investigate the salivary dynamic characteristics of plaque formation around the orthodontic appliances. The aim of the present study was to describe a computational fluid dynamic model to characterize and quantify the salivary flow pattern on labial surfaces of lower central incisors before and following fixed appliances placement to explore the dynamic factors on peri-bracket plaque formation, and, in parallel, to test bacterial amounts on surfaces and periodontal status to assess their relationships.

## Materials and Methods

### Ethics Statement

The study protocol was approved by the institutional ethics board of the Hospital of Stomatology, Sun Yat-sen University. All patients in the study group had provided written informed consent as a part of trial after clinical briefing on methodology.

### Subjects

Twenty-seven patients (17 female and 10 male subjects) scheduled for fixed orthodontic treatment in the Orthodontic Department of Hospital of Stomatology, Sun Yat-sen University were selected for the present study. The mean age of the sample was 21.93±5.50 years (minimum = 11 and maximum = 36). Inclusion criteria were as follows: (1) permanent dentition, (2) no active caries lesions, no staining, no enamel defect, or no initial caries lesion on lower central incisors that were planned to be investigated, (3) normal salivary flow rate (>1.0 mL/min), (4) normal buffer capacity (final pH: 6.5–7.2), and (5) regular tooth brushing habits. Subjects who had used antibiotics during the three-month period prior to the study were excluded. An informed consent form was signed by the patient or the parent before the investigation began. All patients were told to maintain normal dietary and oral hygiene habits, supplied with standardized toothpaste and asked to refrain from any other oral hygiene products for the duration of the trial.

### Clinical Procedures

Fixed orthodontic treatment was performed with directly bonded metal brackets (MBT bracket, Victory, 3M Unitek, Monrovia, CA, USA), applied on incisors, canines and premolars using light cure adhesive, and orthodontic bands cemented with glass-ionomer cement on the first molars. A 0.014-inch nitinol archwire was used for initial leveling one week following appliance bonding. Brackets on the lower central incisors were not ligated and other brackets were ligated with conventional stainless steel ligature wires.

### Microbial and Periodontal Measurements

Microbial and periodontal records were obtained before bonding (T0), one week following bonding (T1), and one week after archwire placement (T2).

**Figure 1:**
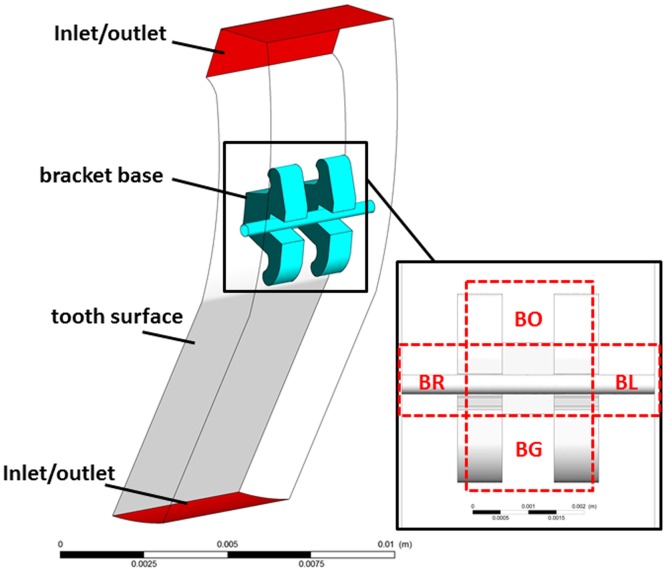
The three-dimensional finite volume model of the lower central incisor with bracket and archwire. The peri-bracket sites were divided as extending 2 mm around the bracket base. Occlusal regions along the bracket (BO), gingival region along the bracket (BG), left region along the bracket (BL) and right region along the bracket (BR).

At each appointment, microbial samples taken from the labial surfaces of the lower central incisors were cultivated and analyzed by the same examiner. The collections were made at the bracket area, defined as extending 2 mm around the center of clinical crown at T0 and 2 mm around the bracket base and divided into four dimensional regions (Figure 1) at T1 and T2. The plaque sample was placed in 1 ml Stuart transport media. Serial 10-fold dilutions of the transport media with the sample of plaque were inoculated on a non-selective medium. Aliquots of 50 µm of the dilutions were inoculated onto non-selective blood agar plates supplemented with 7% sterile sheep blood. The blood agar plates were incubated at 37°C for 3 days in a CO_2_ atmosphere, after which the total number of CFU was counted (the CFU was counted, in four quadrants, if its number was <300).

Periodontal measurements were recorded from all bonded mandibular teeth, but only the scores of the central incisors were considered. The same examiner evaluated periodontal status using a Williams periodontal probe. The gingival index (GI) and pocket depth (PD) values were used for periodontal evaluation.

### Numerical Procedure

Ignoring gravity, three-dimensional governing equations for incompressible and viscous fluid were adopted for mass and momentum conservation.
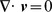


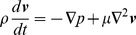
where *v* is the velocity vector, *ρ* is the fluid density, *p* is the pressure, *μ* is the dynamic viscosity.

Tooth surfaces of the lower incisors were reconstructed from CT data with the Materialise's interactive medical image control system (Mimics, Materialise, Belgium), while the bracket models and a 0.014-inch round archwire used in the simulation were built with AutoCAD (Autodesk Inc., CA, USA) according to the clinical bracket dimensions, with a width scale of d = 2.4 mm. The models were meshed using a commercial pre-processing package, GAMBIT (ANSYS Inc., NH, USA), with tetrahedron mesh. The total mesh of each model was ∼500,000.

In this study, the calculation simulated saliva flew on the tooth surface gingivally and occlusally by the swallowing movement, only considering the effect of the bracket to the salivary flow-field. Therefore, the model assumed that the computational domain was rigid and filled with saliva; the surfaces of the tooth and bracket were frictionless; the saliva entered and exited through the upper boundary smoothly with the flow rate represented by a sine curve. The total volume of the saliva in the calculated domain was 0.14 ml and the movement duration was 0.25 s. Saliva density was 1 g/cm^3^ [17].

The models were imported to FLUENT (ANSYS Inc.) for solving the conservation equations [18]. Turbulence was calculated using the RNG k-ε model. The numerical scheme for solving the flow-fields was a pressure-based segregated algorithm, SIMPLE, with aggregative algebraic multigrid (AAMG) method. The calculation was performed with double precision for accurate results.

### Statistical Analyses

All bacterial counts were log 10-transformed for ease of statistical calculations. Mean and standard deviations of the bacterial counts, GI, and PD values were calculated. Paired *t*-test was used to compare mean bacterial counts and PDs of the groups. A Wilcoxon signed rank test was used to compare GI values of the groups. *P*<0.05 was considered statistically significant. All tests were performed using SPSS v11.0 (SPSS, Inc., Chicago, III).

## Results

**Figure 2:**
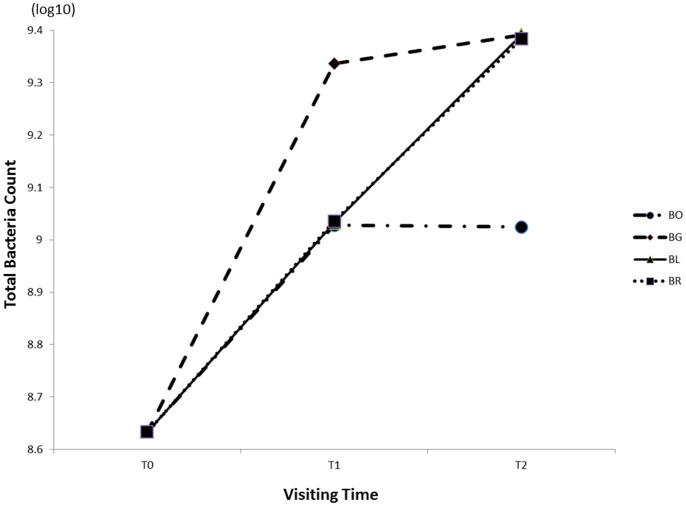
Mean numbers of total colony-forming units (CFU), with a logarithmic scale, at T0, T1 and T2. Four peri-bracket sites includes: occlusal regions along the bracket (BO), gingival region along the bracket (BG), left region along the bracket (BL) and right region along the bracket (BR).

Before and after the application of appliances, the bacteria collected from the tooth surfaces were cultured in blood agar with the colony appearance of 2–3 mm yellowish round shapes. At baseline, the mean value of bacterial counts on the bonded teeth was 8.6332±0.0207. The counts showed similar changes in all sites of the examined teeth that each of the bracket or archwire visit values were found to be significantly higher (*P*<0.01) than each of the first visit means, with mean values of total four sites of 9.1082±0.0056 at T1 and 9.2977±0.0102 at T2. While referring to each specific site, bacterial counts showed similar change principles of increasing trend during the periods from T0 to T2 (Figure 2), except that the bacteria at the BO site was not significantly different between T1 and T2 (*P* = 0.405). It was seen that at T1 or T2, the maximum total bacterial count was both recorded in the region of BG, 9.3366±0.0057 at T1 and 9.3908±0.0177 at T2 (Table 1).

**Table 1:** Amount of total bacteria colony-forming units (CFU) per site at T1 and T2.

	T1			T2		
	CFU	p-value			CFU	p-value
Average per site						
BO	9.0279±0.0118		9.0248±0.0158			
BG	9.3366±0.0057		9.3908±0.0177			
BL	9.0324±0.0096		9.3916±0.0170			
BR	9.0360±0.0131		9.3837±0.0170			
Differences between sites						
BO-BG	−0.3087±0.0115	0.000			−0.3660±0.0198	0.000
BO-BL	−0.0045±0.0137	0.099			−0.3668±0.0218	0.000
BO-BR	−0.0081±0.0189	0.037			−0.3589±0.0232	0.000
BG-BL	0.3042±0.0074	0.000			−0.0008±0.0234	0.870
BG-BR	0.3006±0.0152	0.000			0.0071±0.0226	0.111
BL-BR	−0.0036±0.0167	0.283			0.0079±0.0205	0.055

The first part displays the averages per site, the second part the differences between the sites with the corresponding *P*-values.

Due to the close relationships between dental plaque and periodontal health, patients’ periodontal status was monitored during the entire study period (Table 2). The GI values were very similar to microbiological parameters with a statistically significant increase between T0–T1, T0–T2 and T1–T2 (*P*<0.05). No significant differences in PDs of bonded teeth were determined between the one-week intervals from T0 to T1 and T1 to T2. However, statistically significant differences were detected between the two-week intervals from T0 to T2 (*P* = 0.033).

**Table 2:** Longitudinal changes in periodontal measurements of bonded teeth.

	T0		T1		T2		Significance Between		
	Mean	SD	Mean	SD	mean	SD	T0–T1	T0–T2	T1–T2
Gingival index	0.2222	0.2229	0.2870	0.2471	0.3611	0.2532	0.008	0.001	0.020
Pocket depth	0.6488	0.1755	0.6759	0.1677	0.7203	0.2206	0.230	0.033	0.100

**Figure 3:**
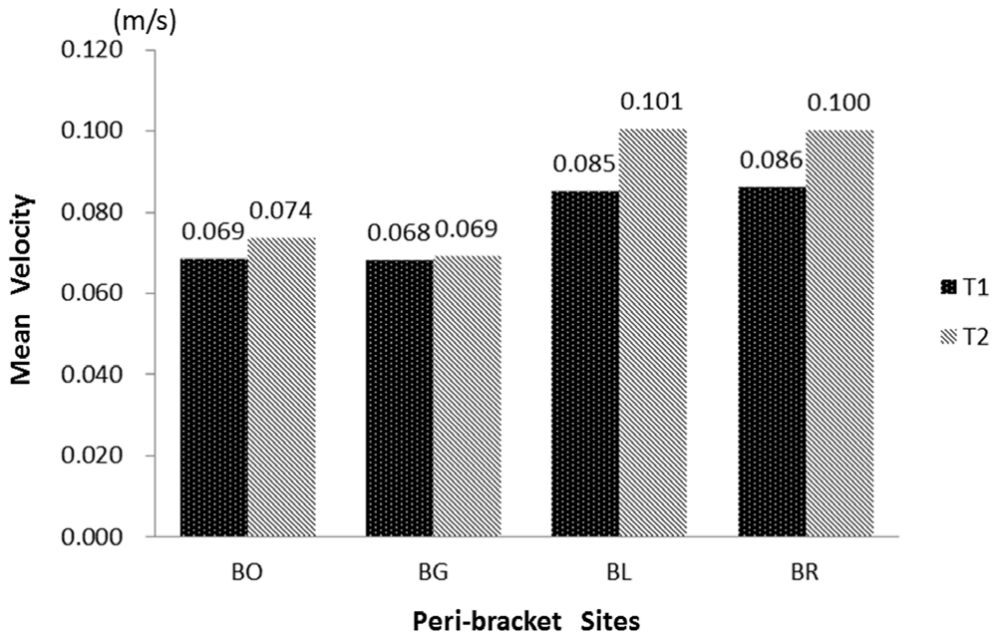
Average salivary velocity of peri-bracket sites at T1 and T2. Occlusal regions along the bracket (BO), gingival region along the bracket (BG), left region along the bracket (BL) and right region along the bracket (BR).

**Figure 4:**
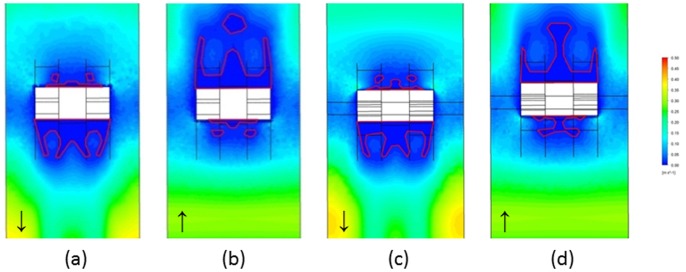
Salivary velocity distribution on lower central incisor. The salivary velocity distribution were displayed when (a) the saliva was flowing gingivally at T1; (b) the saliva flowing occlusally at T1; (c) the saliva flowing gingivally at T2; (d) the saliva flowing occlusally at T2. The red enclosed area illustrated the low velocity areas, and the arrow indicates the direction of saliva flow.

**Figure 5:**
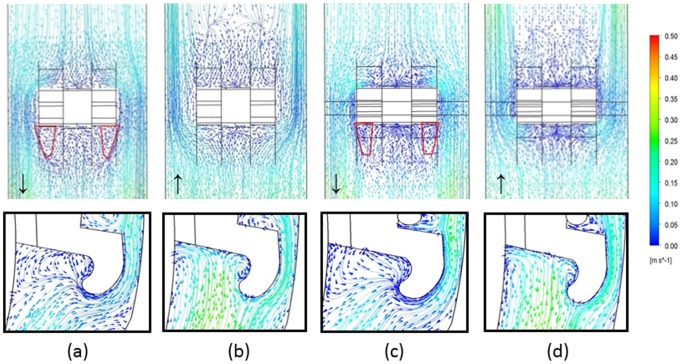
Salivary velocity contour on lower central incisor. The salivary velocity contour were displayed when (a) the saliva flowed gingivally at T1; (b) the saliva flowed occlusally at T1; (c) the saliva flowed gingivally at T2; (d) the saliva flowed occlusally at T2. The bottom figures demonstrated the routes of salivary flow in the gingival region along the bracket (BG). The red enclosed area illustrated the vortex areas, and the arrow indicated the direction of saliva flow.

Based on the dimensions of the bracket and 0.014 inch archwire, we established a 3D model of orthodontic appliances on the lower central incisor surfaces (Figure 1) and simulated the process of saliva flowing over the tooth surfaces fitted with the orthodontic appliances. At a rate of 0.172 m/s, the salivary flow was found to be interrupted by the bracket thereby resulting in a reduction in its flow speed along the bracket bases. The average velocity of salivary flow in the peri-bracket regions is demonstrated in Figure 3 with higher speeds (0.085–0.086 m/s at T1 and 0.100–0.101 m/s at T2) observed in the two lateral sites around the bracket. In the flow field on the tooth surface, the low velocity areas (defined as ≤*v*_max_/100 = 0.005 m/s = 5 mm/s) were present and mainly located near the occlusal and gingival regions around the bracket (Figure 4) and the average size values of low velocity areas were (1) at T1, BO 2.8858 mm^2^, BG 2.3040 mm^2^; (2) at T2, BO 2.3386 mm^2^, BG 2.3213 mm^2^, taking both, gingival and occlusal salivary flow into consideration together. In the low velocity areas, vortexes were formed in the specific BG area only when the saliva was flowing gingivally (Figure 5). The size values for the vortexes were 1.9296 mm^2^ and 1.7683 mm^2^ for T1 and T2, respectively.

## Discussion

Wearing orthodontic appliances complicates the oral environment and restricts the flow of food and saliva on vestibular surfaces due to the contours formed on the smooth surfaces. The surface irregularities protect colonized bacteria from natural removal forces such as salivary flow and muscle action, which may be why rough surfaces are directly associated with increased bacterial accumulation and higher incidences of gingival inflammation. Nevertheless, high fluid shear or pressure loading could upregulate the expression of gingival overgrowth related factors, such as MMP8 [19], MMP9 and collagen IV [20], providing a shelter for bacterial accumulation under the hypertrophied gingiva, ultimately culminating in alterations in the structure and function of the periodontal tissues. The objective of the present study was to investigate salivary dynamic characteristics of plaque retention and periodontal status around appliances during orthodontic treatment.

In a swallowing cycle regulated by muscle movements and pressure changes, saliva from various glands enters the mouth, gradually fills the vestibule and then flows through the dentition gap to the oral cavity proper [21]. Within the relatively close space in the vestibule limited by the lips, vestibular groove and labial surfaces of the teeth, saliva flows were interrupted by the placement of bracket and archwire with a typical pattern of flowing on the tooth surface, then lashing against the gingival or occlusal margins of the brackets with small vortexes and afterwards splitting into two channels along the bracket contours on the bilateral sides (Figure 5). When the saliva flows along the bracket base, the flow rate is decreased as shown in Figure 4. With lower velocity, the clearance effect of saliva will be reduced, and the bacteria could colonize on the tooth surfaces that have few bacteria agglomerated in appliance-free conditions [22]. In our microbiologic study, the total bacterial counts significantly increased from 8.6332±0.0207 at baseline to 9.1082±0.0056 at T1 and 9.2977±0.0102 at T2, which is in good agreement with previous studies [23] suggesting that orthodontic appliances result in an increase in bacterial numbers, and demonstrates the predictions of the effect of fluid velocity on bacteria retention.

Although total bacterial counts on the tooth surface increased with the placement of orthodontic appliances, differences exist between the sites. The present results of site-specific bacterial accumulation demonstrate that in areas gingival and occlusal to the bracket with lower salivary velocity, a greater increase in bacterial counts was detected when compared to the bilateral sites (Figure 3), The sizes of these two low velocity areas were similar (2.3040–2.8858 mm^2^ at T1 and 2.3213–2.3386 mm^2^ at T2). However, the counts in the gingival areas were significantly higher, irrespective of the time periods T1 or T2 (*P*<0.001), which may be attributed to two factors. The first is the poor cleansing action wherein the narrowness of the gingival region located between the gingival tissues and bracket due to the muscle strength of orbicularis oris and depressor labii inferioris covering the incisors makes it difficult for the toothbrush to reach. Additionally, the rough labial mucosa produces a negligible effect on tooth surfaces cleaning, weakening the self-cleansing behavior of the oral cavity. The second is the formation of vortexes, which occurred only in the area gingival to the bracket. In vortexes, saliva flows in a spiral motion at a flow rate approaching zero (Figure 5), dramatically increasing the salivary clearance half-time and allowing the diffusant to accumulate over the plaque, as the glycoprotein in saliva provides binding sites for bacterial adhesion, thus indicating the reason as to why demineralization sites commonly locate in the gingival portion of the bracket base [23].

Following archwire placement, a slight increase in velocity was observed locally in the peri-bracket areas when compared to the bonding visit (Figure 3), yet bacterial counts were found to increase from T1 to T2 (Figure 2). In the presence of the archwire, the space for the same volume of saliva flowing on the tooth surface was reduced, and thus the saliva, flows at higher rate especially beneath the wire and with a complicated flow field when compared with a situation where only the bracket is placed. The limited space of an archwire could increase the difficulty and decrease the effect of tooth brushing in this region, consecutively increasing the number of bacterial colonies, explaining the clinical observation in BL, BR and BG areas in this study. As for the BO site, archwire placement did not affect the bacterial counts (*P* = 0.405), due to the wide smooth surface in this area and ease of cleansing. Therefore, oral hygiene instructions such as movement of the toothbrush at various angles to clean the surfaces beneath the archwire and beside the bracket effectively, before orthodontic treatment, are strongly recommended.

With the accumulation of microbial flora, a certain deterioration of the gingival and periodontal status has been reported in orthodontically-treated patients. In our study, results of the periodontal evaluation revealed a significant increase in the GI value, showing similar trends with bacterial counts in the BG area. This increase is in agreement with the results of numerous studies that suggest that fixed orthodontic appliances lead to an increase in gingival inflammation. In the present study, significant differences (*P* = 0.033) in PDs of bonded teeth were determined between T0 and T2, perhaps as a result of a lengthy observation period of the destruction of attached gingival. This was in disagreement with Huser’s report, which suggested that the probing depth in both orthodontic and control groups remained within normal values. The origin and pathogenesis of periodontal diseases are known to be multifactorial, but dental plaque certainly is an essential precursor [11]. The placement of orthodontic brackets does create new locations for plaque retention, especially at gingival portions of the tooth surface, thereby increasing the inflammatory response. To prevent detrimental effects of orthodontic treatment on periodontal and gingival tissues, oral hygiene programs are needed, especially the gingival areas surrounding the brackets.

Following placement of the bracket and archwire, the tooth surface was almost completely covered by a thick layer of bacteria, with various changes in different dimensional areas surrounding the bracket base. This study confirms that saliva flow interrupted by the appliance is an obvious predisposing factor for bacterial accumulation due to its low velocity and vortexes. Although, undoubtedly, there are limitations to this model considering the highly complex nature of the multiple factors that influence bacterial adhesion to solid surfaces, this study allows us to replicate *in* *vitro*, the salivary characteristics of plaque retention. However, further work is required to refine and extend this model.
